# Sensitive and quantitative detection of cardiac troponin I with upconverting nanoparticle lateral flow test with minimized interference

**DOI:** 10.1038/s41598-021-98199-y

**Published:** 2021-09-21

**Authors:** Sherif Bayoumy, Iida Martiskainen, Taina Heikkilä, Carita Rautanen, Pirjo Hedberg, Heidi Hyytiä, Saara Wittfooth, Kim Pettersson

**Affiliations:** 1 Department of Biotechnology, University of Turku, Turku, Finland; 2 Cancer Research and Translational Medicine Research Unit, Department of Clinical Chemistry, University of Oulu, Kajaanintie 50, 90220 Oulu, Finland; 3 The Joint Municipal Service Provider of Northern Finland Laboratory Centre (NordLab), Kiviharjuntie 11, 90220 Oulu, Finland

**Keywords:** Biochemical assays, Diagnostic markers, Myocardial infarction, Blood proteins

## Abstract

Measurement of cardiac troponin I (cTnI) should be feasible for point-of-care testing (POCT) to diagnose acute myocardial infarction (AMI). Lateral flow immunoassays (LFIAs) have been long implemented in POCT and clinical settings. However, sensitivity, matrix effect and quantitation in lateral flow immunoassays (LFIAs) have been major limiting factors. The performance of LFIAs can be improved with upconverting nanoparticle (UCNP) reporters. Here we report a new methodological approach to quantify cTnI using UCNP-LFIA technology with minimized plasma interference. The performance of the developed UCNP-LFIA was evaluated using clinical plasma samples (n = 262). The developed UCNP-LFIA was compared to two reference assays, the Siemens Advia Centaur assay and an in-house well-based cTnI assay. By introducing an anti-IgM scrub line and dried EDTA in the LFIA strip, the detection of cTnI in plasma samples was fully recovered. The UCNP-LFIA was able to quantify cTnI concentrations in patient samples within the range of 30–10,000 ng/L. The LoB and LoD of the UCNP-LFIA were 8.4 ng/L and 30 ng/L. The method comparisons showed good correlation (Spearman’s correlation 0.956 and 0.949, p < 0.0001). The developed UCNP-LFIA had LoD suitable for ruling in AMI in patients with elevated cTnI levels and was able to quantify cTnI concentrations in patient samples. The technology has potential to provide simple and rapid assay for POCT in ED setting

## Introduction

Cardiac troponin I (cTnI) has been used clinically, for years, for diagnosis and risk stratification in patients with suspected acute myocardial infarction (AMI)^1^. Generally, elevated levels of cTns are conjoined with damage of cardiac muscles and cTnI release to the circulation has specificity for cardiac injury^2^. Measurement of circulating levels cTnI, particularly observing the rise or fall in cTnI levels, along with the evaluation of patient symptoms, history and electrocardiographic (ECG) abnormalities are current procedures in the triage of suspected AMI patients^3,4^. The typical challenge is early identification of AMI among the large heterogenous patient population, as only one in three patients, experiencing chest pain, who visit emergency department (ED) receives AMI diagnosis^5^. Therefore, timely and correct diagnosis to rule-in AMI is important in terms of cost savings and improving the clinical outcomes of AMI patients.

Point-of-care testing (POCT) can facilitate quick turn-around-times (TAT) to promote efficient diagnosis of AMI^6^*.* Lateral flow immunoassays (LFIAs) can be used in POCT, particularly in settings where the cost of the high-tech instrumentation may be prohibitive. LFIA in POCT is well-established, mature technology known for its low cost. However, the limitations regarding sensitivity and quantification have hindered its use in many applications^7^*.* In our previous work, we have described how the use of upconverting nanoparticle (UCNP) reporters in LFIAs provides quantitative results and improved sensitivity^8–10^*.* The use of UCNP measurement technology can enhance the assay sensitivity in comparison to traditional down-converting fluorescent reporters since it enables elimination of the background autofluorescence from the measurement. This is due to the unique photon upconversion luminescence of UCNP reporters converting low-energy excitation (near or at infrared) wavelength into high-energy emission at visible wavelengths^11^. The UCNP signal can be read with a miniaturized relatively low-cost reader instruments with high performance in POCT settings. Such readers with miniaturized optics for UCNP measurement have previously been described in the literature^12–15^.

The performance of LFIAs may suffer from interferences originating from the sample matrix, particularly because of the typical one or two step assay procedure with limited washing. These immunoassay (IA) interferences may be caused by different interfering agents such as circulating heterophilic IgG and IgM antibodies^16^, autoantibodies targeted against the biomarker of interest^17^ and human anti-animal antibodies^18^. In general, IgG antibodies used as binders in IAs are prone to several interfering reactions caused by circulating antibodies. If a heterophilic antibody has high affinity for the Fc-region of an IA binder antibodies, an aggregate of binder antibodies with large numbers of Fc-regions close together functions as a binding target for the heterophilic antibody^16^. Furthermore, it has been shown that complement activation by the classical pathway can occur when several IgG molecules are in close proximity, e.g. on reporter nanoparticle surface^19^ or on the solid phase^20^. As a results, complement factors bind to the Fc region of the IA binder antibody^20,21^ which causes negative interference in the IA. Non-specific binding of autoantibodies such as rheumatoid factor (RF) has been associated with falsely elevated analyte concentrations^22^. Since IAs utilizing nanoparticle reporters aim at extremely high sensitivities, the effect of matrix interferences should be addressed to fully utilize the potential of nanoparticle IA technologies.

It has been depicted that the effects of interfering agents can be eliminated with sample pre-treatment procedures such as heating or precipitation of interfering antibodies with polyethylene glycol (PEG)^20^. In addition, pretreatment with Ethylenediamine tetra-acetic acid (EDTA) effectively inhibits the activation of the complement pathway^23^. However, particularly in the case of POCT, time-consuming sample pre-treatment steps are undesirable. Therefore, in LFIAs the potential solutions should be implemented in the test device itself.

In this study, we report the development of a UCNP-LFIA for cTnI. UCNP reporter technology was utilized to provide the sensitive and quantitative detection of cTnI in LFIA format to overcome the typical limitations of LFIAs. The matrix interference in plasma samples was studied and reduced by incorporating the developed sample pre-treatment steps into the LF strip. The performance of the developed UCNP-LFIA was evaluated with clinical plasma samples (n = 262).

## Results

### Elimination of matrix interference

**Figure 1:**
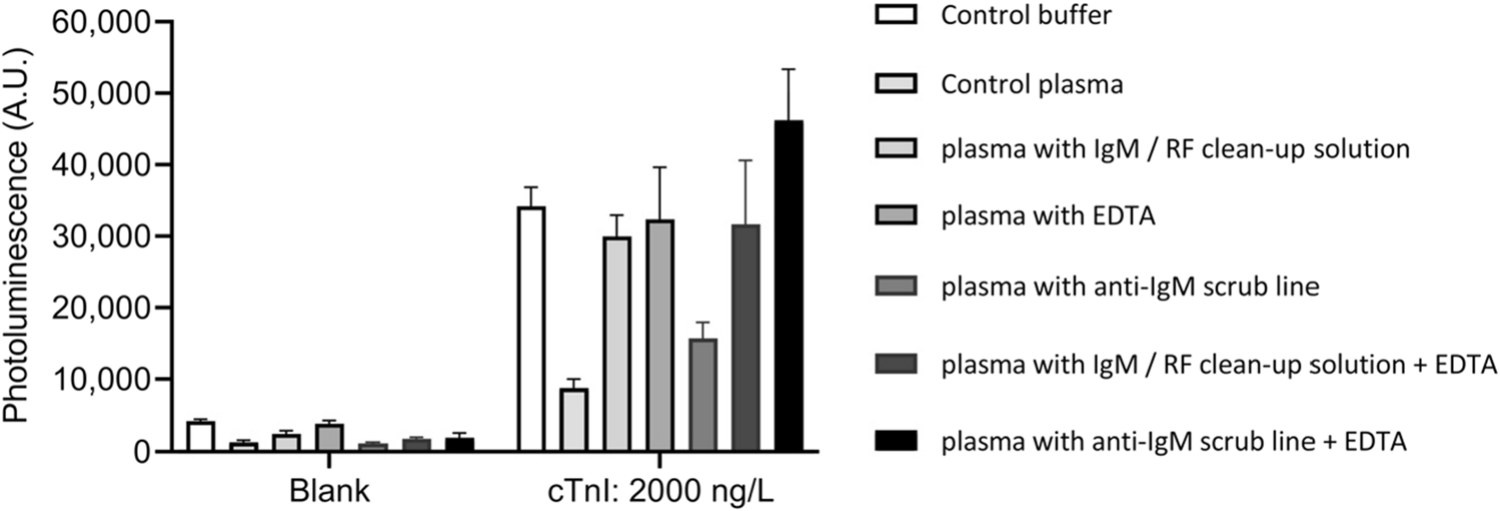
The effect of sample pre-treatment on plasma interference. All samples were analysed as unspiked (blank) and spiked with 2000 ng/L of cTnI before the pre-treatment. Pooled EDTA plasma samples were used in this experiment. IgM and RF commercial clean up solution was compared to 250 mM EDTA, IgM scrub line (prior to test line) and to different combinations. The error bars represent standard deviations of three replicate LFIA strips.

In LFIA strips constructed without a scrub line and EDTA in the sample pad, the addition of cTnI to LiH plasma samples led to significantly lower signal recovery as compared to what was seen with 7.5% BSA-TSA buffer. These results implied that plasma contains substances which interfere with the performance of the assay. To avoid this interference various sample pretreatment options were tested, and the results are shown in Fig. 1. What stands out in the results is the enhancement of signal levels by all the tested pretreatment steps. While untreated plasma showed a calculated recovery of 24%, the pretreatment steps produced notable improvement in recoveries reaching values around 60–89%. LFIA strips with a scrub line and EDTA treatment induced around fivefold improvement in signals compared to the untreated LiH plasma and a 136%, recovery. As a result, anti-IgM scrub line and EDTA in the sample pad were included in all LFIA strips in the following experiments.

### Calibration curve, Limit of detection, and linearity

**Figure 2:**
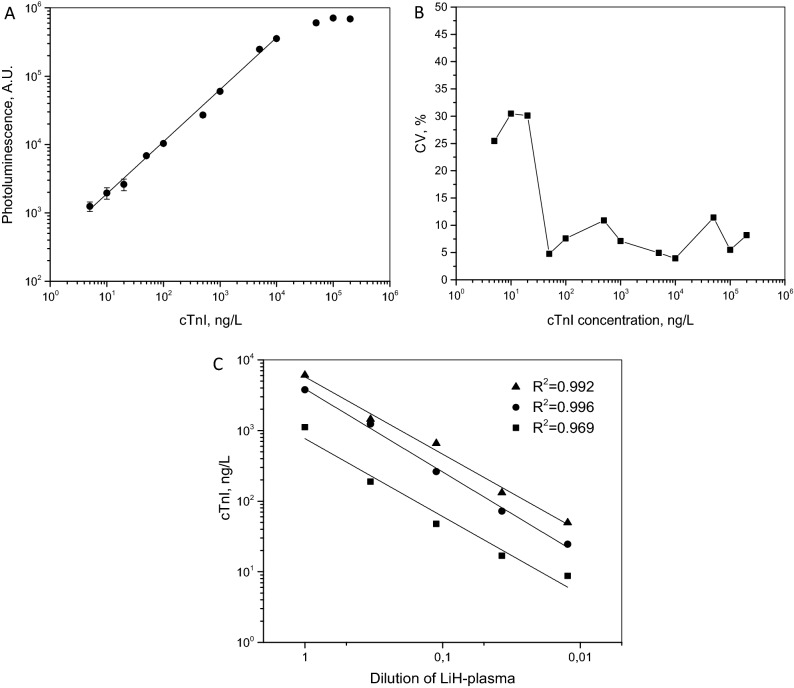
Analytical performance of the developed proof of concept cTnI LF test, showing the standard curve, precision profile and parallelism. (**A**) Calibration curve for the UCNP-LFIA. The error bars represent the standard deviation of three replicate strips. Equation of the curve was y = 0.76x + 2.52, R2 = 0.996. (**B**) Precision profile determined from the calibration curve measurements. CV% describes the variation between the calculated cTnI concentrations. (**C**) Parallelism of the UCNP-LFIA. Three clinical LiH-plasma samples containing variable amounts of endogenous cTnI were serially diluted to 1/81-fold with blank LiH-plasma pool.

A calibration curve for the UCNP-LFIA (in the final format with the anti-IgM scrub line and EDTA supplemented sample pad) is shown in Fig. 2A. The curve was linear up to 10,000 ng/L (y = 0.76x + 2.52, R^2^ = 0.996). The LoB of the assay was calculated to be 8.4 ng/L and the LoD was 30 ng/L. The linearity of the UCNP-LFIA was studied with serial dilutions of three patient samples diluted by 3 to 81-fold using blank LiH plasma pool (Fig. 2B). The linear regression of the observed cTnI concentration and dilution factor showed good linearity R^2^ = 0.969–996 over the measured range of 37–9 365 ng/L (Fig. 2C).

### Method comparison

**Figure 3:**
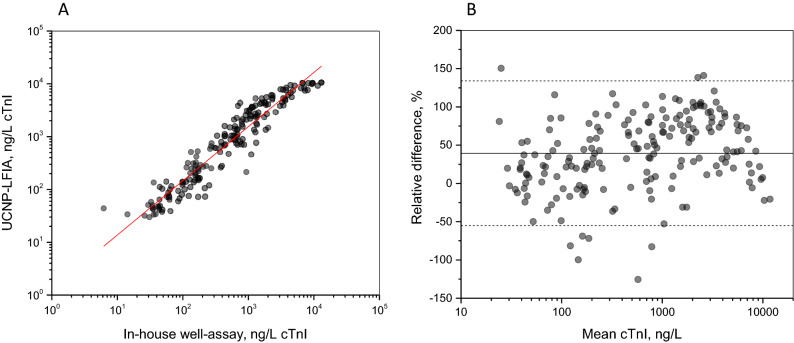
Method comparison between the UCNP-LFIA and the in-house well-based reference assay (n = 188). (**A**) Correlation of the assays and (**B**) Bland–Altman analysis of agreement. The relative difference is calculated as UCNP-LFIA concentration subtracted by in-house well-based assay concentration divided by mean concentration. The mean difference (39.4%) is presented with a solid line and the 95% limits of agreement (from − 55.1 to 134%) are shown with dashed lines.

Method comparisons were conducted using LiH-plasma samples with varying cTnI concentrations (n = 262). Samples below the LoD and outside the linear range of the assays were excluded from the comparisons. The method comparison between the UCNP-LFIA and the in-house well-based assay (n = 188) is shown in Fig. 3A. Passing and Bablok regression analysis for the UCNP-LFIA and the in-house well-based assay resulted in a slope of 1.11 (95% CI from 1.05 to 1.16) and a y-intercept of − 0.08 (95% CI from 0.02 to 0.05). The Spearman correlation coefficient was 0.956 (p < 0.0001). The mean relative difference between the two methods was 39.4% with the 95% limits of agreement ranging from − 55.1 to 134% (Fig. 3B). A close-up on the correlation between the developed LFA and the in-house well-based assay within the cTnI concentration range of less than 100 ng/L is shown in Fig. S1.

**Figure 4:**
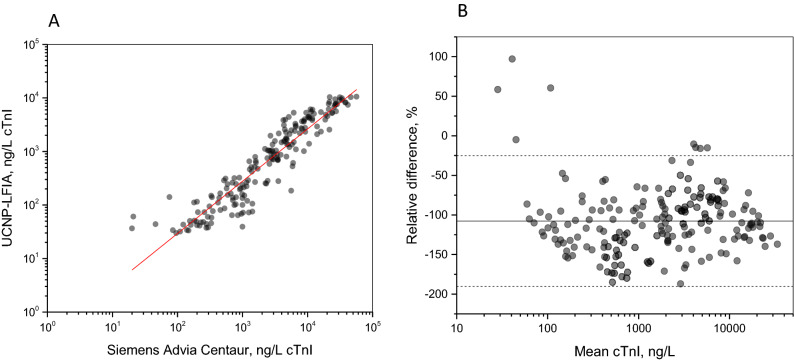
Method comparison between the UCNP-LFIA and Siemens Advia Centaur TnI-Ultra reference assay (n = 191). (**A**) Correlation of the assays and (**B**) Bland–Altman analysis of agreement. The relative difference is calculated as UCNP-LFIA concentration subtracted by Siemens Advia Centaur TnI-Ultra concentration divided by mean concentration. The mean difference (− 107.7%) is presented with a solid line and the 95% limits of agreement (from − 25.1 to − 190.4%) are shown with dashed lines.

Method comparison between the UCNP-LFIA and the Siemens Advia Centaur assay (n = 191) is shown in Fig. 4A. Passing and Bablok regression analysis for the UCNP-LFIA and Siemens Advia Centaur yielded in a slope of 1.07 (95% CI from 1.02 to 1.13) and a y-intercept of − 0.79 (95% CI from − 0.98 to − 0.63). The Spearman correlation coefficient was 0.949 (p < 0.0001). The mean relative difference between the two methods was − 107.7% with the 95% limits of agreement ranging from − 25.1 to − 190.4% (Fig. 4B).

## Discussion

In this study, we developed an LFIA utilizing UCNP reporters to enable sensitive and quantitative detection of cTnI in LiH plasma samples with minimized interference. The UCNP-LFIA was able to quantify cTnI concentrations in patient samples within the range of 30–10,000 ng/L with a read-out time of 30 min. By introducing an anti-IgM scrub line and dried EDTA in the LFIA strip, the detection of cTnI in plasma samples was fully recovered. Measurement of cTnI levels in point-of-care (POC) setting requires rapid assays with high sensitivity to determine whether an acute myocardial infarction (AMI) has occurred. Despite the advances in POCT technologies, detecting cTnI is still particularly challenging since cTnI assays are prone to interferences originating from biological compounds such as circulating antibodies^24^. Ideally, a POCT device for cTnI would confront these challenges, be usable outside of clinical laboratories and enable desired sensitivity and quantification. Therefore, implementation of simple, easy-to-use tests such as lateral flow tests in POCT settings could be feasible as the lateral flow platform can be adapted with minimal training and surrounding laboratory infrastructure.

The presence of autoantibodies and other interfering compounds in the sample matrix are common problems in immunoassays resulting in loss in the detection sensitivity. Here we describe approaches to reduce these kinds of interferences in an LFIA. The developed UCNP-LFIA utilized 3 + 1 configuration of Mab binders for the detection of cTnI. The selected configuration reduces the cTnI autoantibody effect as described previously^25,26^. However, cTnI IAs are still prone to interfering effects of other biological components present in blood. IA interferences have been shown to originate from e.g., the binding of IgM and complement factors to the IA binder antibodies thus preventing the signal generation^20,21,23^. Furthermore, human RF exists in its predominant forms of IgM and IgG possibly resulting in falsely elevated analyte concentrations^22^. Therefore, we investigated the effect of removal of both immunoglobulin types IgM and IgG from the LiH plasma. A profound improvement in signal levels and recoveries in the treated samples was observed in comparison to the untreated samples. However, the IgM/RF removal pretreatment requires more than 2 h to perform, which prevents its use in a POCT solution. Therefore, we added an EDTA treated sample pad and anti-IgM antibody scrub line dispensed on the nitrocellulose membrane to the LFIA test strips. These techniques incorporated to the test strips improved the signal recoveries without a separate pretreatment step. The findings provide valuable information on improving the analytical and clinical performance of the LFIA test devices also in general.

To overcome the limitations of the conventional LFIA technology in cTnI detection, we used UCNP reporters to generate a sensitive and quantitative assay in LFIA format. The UCNP reporters have been previously shown to enable these desired properties in LFIA applications^27,28^. In addition to the quantitatively measurable upconversion photoluminescence signal, the UCNPs can be used for more sensitive applications. The measurement sensitivity increases since the photon upconversion originating from the UCNP reporters can be spectrally separated from the down-converting background autofluorescence^11^. For the developed UNCP-LFIA, the obtained LoD was 30 ng/L. The method comparison to the two reference assays suggests that the UCNP-LFIA could be used for the quantitative determination of cTnI. Typically, low and moderate cTnI levels around 50–100 ng/L and temporal increase in cTnI level of more than 15 ng/L in the subsequent measurements are suggestive for AMI^29^. The developed LFIA concept showed potential to be used for the detection and monitoring of elevated cTnI levels in suspected AMI patients. Analytically, the developed LFIA showed a wide range of linearity, good parallelism response and precision profiles.

The assay procedure of the UCNP-LFIA was simple with a TAT of 45 min. POCT for cTnI in the EDs has been shown to reduce the length of stay for patients with suspected AMI^30^. Accelerated diagnostic protocols for AMI use serial cTnI testing with subsequent measurements of cTnI concentration to determine possible elevation in circulating cTnI concentration^29^. Blood samples for the measurement of cTnI should be drawn on first assessment and repeated 3–6 h later^3,4^. Thus, the results should be available within one hour TAT. To consider implementation of the developed UCNP-LFIA concept to the POCT use in clinical routine, the pre-incubation step should be integrated to the LF test device to provide a user-friendly POCT assay. Furthermore, reducing TAT from 45 min to less than 30 min would improve the UCNP-LFIA feasibility for POCT^31^. The ability to test whole blood samples would further facilitate the POCT use of the LFIA test device.

In contrast to the Siemens Advia Centaur reference assay, the UCNP-LFIA systematically gave lower cTnI values. This bias was also previously observed between the in-house well-based assay and the Siemens Advia Centaur TnI-Ultra reference assay^32^. Antibodies targeting different epitopes in different assays may result in differences in the measured cTnI values^25^. In addition, differences may arise from differences in calibration as standardization of cTnI assays has not been successful^33,34^. The similarity between the UCNP-LFIA and the in-house well-based assay can be explained by the fact that these assays share a very similar 3 + 1 antibody design and utilize the same calibration material^32^.

The results of our evaluation show that the developed UCNP-LFIA technology can be used to detect cTnI in plasma in concentrations relevant in AMI diagnostics. The clinical utility of the developed test should be further evaluated with a cohort of healthy controls and with samples from patients with chest pain visiting EDs. Furthermore, LFIA strip manufacturing and reporter drying process is known to be critical for the test performance even in industrial scale^35^. Therefore, the final clinical performance of the test, including the determination proper reference ranges of the UCNP-LFIA, should be evaluated with a finally optimized production prototype of the UCNP-LFIA manufactured with controlled and reproducible processes.

To sum up, detection of cTnI for diagnosis of AMI requires both high sensitivity and accurate quantitation. In this study, we developed a proof-of-concept UCNP-LFIA for sensitive detection of cTnI and demonstrated with patient samples that the UCNP-LFIA technology can be used for this type of demanding quantitative analyses. Such LFIA could be used for aiding in diagnosis of AMI patients at the ED departments in POC settings, particularly in resource-limited settings where central laboratory diagnostics or high-cost POCT instrumentation is not feasible. The sample pretreatment options applied in this study can provide valuable information for LFIA development also for other analytes requiring high analytical and clinical performance.

## Materials and methods

### Clinical samples

The clinical sample panel consisted of leftover lithium heparin (LiH) plasma samples (n = 262) with varying cTnI concentrations. The samples were randomly collected at Oulu University Hospital (Oulu, Finland) in agreement with ethical standards of the Ethics Committee of the University Hospital of Oulu, in 2013. All procedures were in accordance with the Helsinki Declaration as revised in 2006. The frozen samples were thereafter shipped to the University of Turku in 2013 on dry ice and stored frozen at − 70 °C. Prior to the analysis, the frozen samples were thawed at + 23 °C, mixed, and centrifuged (1 min, 2000×*g*) to remove any particulate material. The concentrations in the samples had been determined with two methods prior to this study: Siemens ADVIA Centaur TnI-Ultra (Siemens Healthcare GmbH, Erlangen, Germany) with a limit of detection (LoD) of 9 ng/L^36^ at Oulu University Hospital and an in-house well-based assay with an LoD of 3.3 ng/L^32^ at the University of Turku. Serum and LiH-plasma samples were collected from apparently healthy individuals (n = 5) at the Department of Biotechnology (University of Turku, Turku, Finland) and the samples were stored at − 20 °C before use. These samples were used for preparation of blank serum and plasma pools. Before the LFIA analysis, all the frozen samples were thawed at + 23 °C and vortexed. The LiH-samples were also centrifuged (1 min, 2000×*g*); to remove any particulate material.

### Antibodies and cTnI calibrators

**Figure 5:**
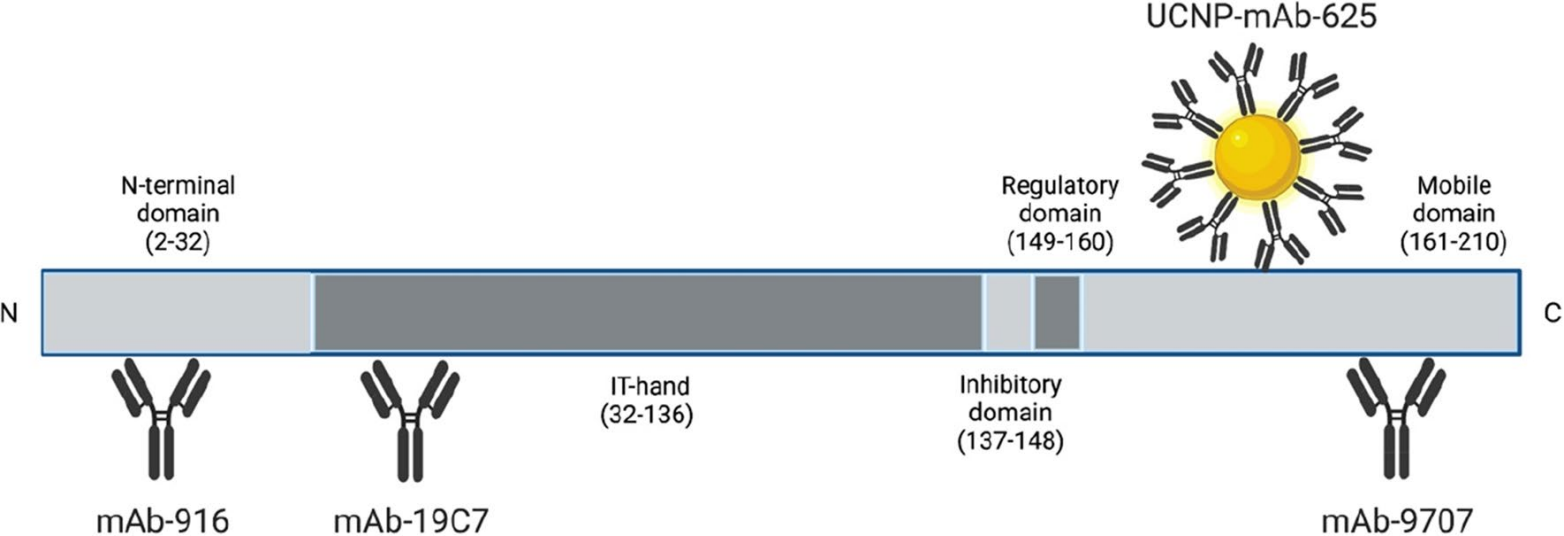
A schematic illustration of the cTnI epitopes and the binding sites of the antibodies in the developed LFIA. The developed UCNP-LFIA utilized the (3 + 1) assay design, with three capture antibodies against the *N*-terminus, midfragment and C-terminus of cTnI and one tracer antibody against the C-terminus of cTnI molecule. Created with www.BioRender.com.

cTnI-specific Mabs 916, 19C7 and 625 (epitopes at residues 13–22, 41–49 and 169–178, respectively) were obtained from HyTest Ltd (Turku, Finland). Anti-h cTnI 9707 SPTN-5 Mab with the epitope in the C-terminal part of the cTnI molecule (residues 190–195) was purchased from Medix Biochemica Oy (Espoo, Finland). The antibody binding on cTnI epitopes is illustrated in Fig. 5. A goat polyclonal anti-IgM (μ-chain specific) antibody (product# I1636) was purchased from Sigma-Aldrich (Saint Louis, MO, USA). Human cardiac troponin complex (I-T-C) (#8T62) was obtained from HyTest Ltd, and the cTnI calibrators were prepared by diluting this complex to blank LiH plasma pool. The blank LiH plasma pool and serum pool from the same individuals was compared to cTnI free serum (HyTest) to confirm signal levels equal to cTnI free serum in both pools.

### Antibody bioconjugation of upconverting nanoparticles

Carboxylated Upcon™ UCNP-reporter particles of 78 nm diameter with a hydrophilic coating (Kaivogen Oy, Turku, Finland) were covalently coupled with the Mab 625. Briefly, the UCNP solution containing 0.5 mg UCNPs was centrifuged for 30 min at 20,000×*g* at room temperature (RT). The supernatant was removed, and the particle surface was activated by suspending the pellet into 135 µL of 50 mM MES containing 2 mM EDC (Sigma-Aldrich) and 30 mM sulfo-NHS (Sigma-Aldrich). The incubation was performed in shaking for 15 min at RT. The UCNPs were washed by centrifugation for 10 min at 20,000×*g* at RT, removing the supernatant and suspending the UCNPs to 20 mM MES, pH 6.1. The UCNPs were centrifuged as before and resuspended into 50 mM MES, pH 6.1 containing 30 µg of Mab 625 and 100 mM NaCl with the total volume of 500 µL. This conjugation reaction was incubated for 30 min at room temperature in shaking and the reaction was stopped by adding glycine, pH 11, to a final concentration of 50 mM. The reaction was further incubated for 30 min at room temperature in shaking. After incubation, the UCNP-conjugates were washed twice to remove unbound compounds by centrifugation for 20 min at 17,200×*g* at + 4 °C and the UCNP pellet was suspended to 250 µL of storage buffer containing 25 mM borate pH 7.8, 150 mM NaCl, 0.1% NaN_3_, 2 mM KF, 0.2% BSA (BSA: Sigma-Aldrich). This wash step was repeated twice and finally the pellet was suspended to 125 µL of storage buffer.

### Preparation of lateral flow strips

The lateral flow test cards were assembled on a plastic support (Standard Grade Backing Laminate, Kenosha Tapes, Amstelveen, Netherlands) by laminating 25 mm wide nitrocellulose printed with the scrub line and the test and control lines, a 34 mm cellulose absorbent pad (CFSP223000, Merck Millipore, MA, USA) and a 16 mm glass fiber sample pad (8950, Ahlstrom-Munksjö Oyj, Helsinki, Finland). The sample pad was pre-treated with 10 mM Borate buffer solution pH 7.5 containing 0.1% Tween-20 and 0.5% casein and 50 mM EDTA. After pre-treatment, the pads were dried at + 35 °C for 2 h. The antibody lines were dispensed in 10 mM Tris–HCl, pH 8.0 in the presence of 5% EtOH and 1% sucrose on the nitrocellulose membrane LFNC-C-BS023 (Nupore Filtration Systems Pvt. Ltd., Ghaziabad, India). The test line solution consisted of Mabs 19C7, 916 and 9707 and the proportions of the Mabs in the dispensing solution were 40%, 40% and 20%, respectively. The test line Mabs were dispensed on the nitrocellulose membrane with the protein density of 1.2 µg/cm. The control line was printed 5 mm from the test line with 0.6 µg/cm of rabbit anti-mouse IgG (Dako Products, Agilent Technologies Inc., Wood Dale, IL, USA). The scrub line was printed 5 mm before the test line location with goat anti-human-IgM IgG antibodies to the density of 0.5 µg/cm. After dispensing the lines, the cards were dried for 2 h at + 35 °C, other components of the test strips were assembled, and the cards were stored at room temperature protected from humidity. The cards were covered with transparent cover tape (KN-CPP1-Clear Kenosha cover plastic, Kenosha Tapes) from sample pad to the end of the absorbent pad. Before the use, the cards were cut into 4.8 mm wide strips.

### cTnI-UCNP lateral flow assay

**Figure 6:**
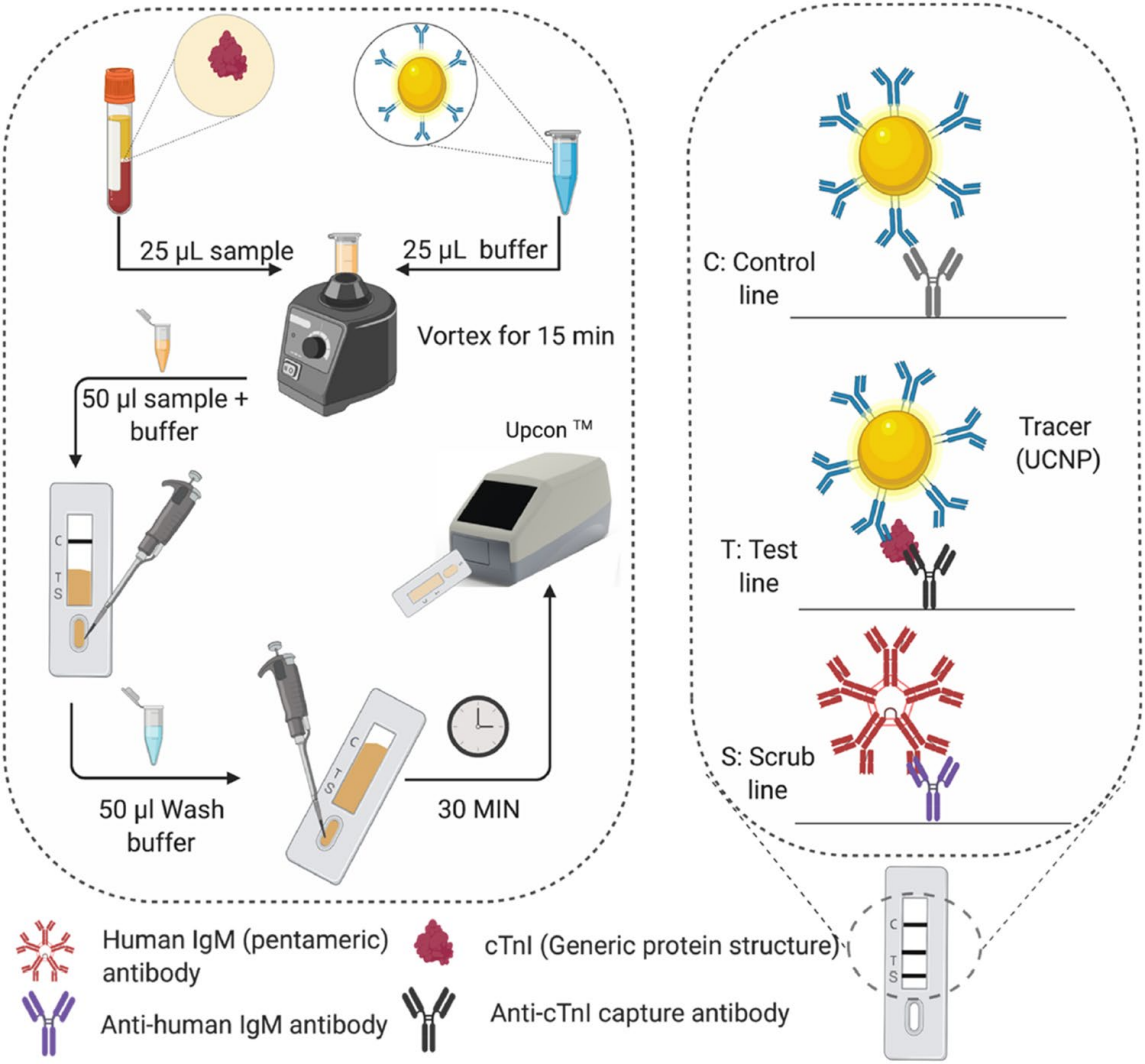
Schematic illustration of the protocol of the developed lateral flow assay and the strip design. Created with www.BioRender.com.

The UCNP-LFIA procedure was initiated by mixing 25 µL of sample and 25 µL of reporter solution (20 ng UCNP diluted in wash buffer solution containing 0.05 M Tris pH 7.5, 0.5 M NaCl, 0.04% NaN_3_, 2 mM KF, 1.5% BSA, 0.06% bovine IgG, 0.2 mg/mL mouse IgG and 0.05 mg/mL denatured mouse IgG) and pre-incubating for 15 min at + 35 °C in slow shaking. First, the total volume of 50 µL of the mixed sample and reporter solution was allowed to absorb to the sample pad of the strip followed by immediate addition of 50 µL of wash buffer. The liquid was allowed to absorb to the strip for 30 min before measurement. The strips were read with an Upcon reader device (Labrox Oy, Turku, Finland). The UCNPs were excited at 976 nm and the emission was measured at 540 nm with a Z-focus point of 8 mm over the range of 12 mm or 125 scanning points. Emission spot size was adjusted at 1 mm for the lateral flow strips. The assay procedure is illustrated in Fig. 6.

### Matrix interference studies

The elimination of interference was first studied with LFIA strips that did not contain the scrub line. The removal of the RF and IgM from the serum/plasma sample prior to addition into the LF strips was investigated by using the commercial clean up solution (ab215121, Abcam, Cambridge, UK). The pretreatment was done as follows: the solution was diluted to the sample in a 1:10 ratio. The samples were incubated at 4 °C for 1 h, prior to centrifugation at 700×*g* for 45 min at 4 °C. The supernatant was carefully transferred to a fresh tube, to avoid any formed pellets. Samples were immediately assayed according to the developed UCNP-LFIA protocol described above. The signal levels obtained from LiH plasma pool spiked with 2000 ng/L cTnI (I-T-C troponin complex) were compared to those obtained from spiked 7.5% BSA-TSA buffer and spiked untreated LiH plasma. The impact of EDTA on the performance was studied by drying 50 mM EDTA to the sample pad. To further investigate the practical means of avoiding the matrix interference, anti-IgM scrub line was added to the LFIA strips as described above.

### Assay evaluations

The limit of blank (LoB) and limit of detection (LoD) for the final assay design were determined according to the Classical Approach of CLSI Guideline EP17-A2 using above described blank LiH plasma pool as a zero calibrator and five LiH plasma pools with spiked cTnI concentrations of 1–4 × LoB. Initial LoB was determined with 20 replicates of blank pool samples. The blank pools and low concentration samples were run during 4 days in 5 replicates each. Due to the non-normal distribution of the blank sample results, a non-parametric data analysis was used. For low concentration samples, the non-parametric data analysis approach was used because of the non-normal distribution among the results from these samples. In the non-parametric analysis, LoD was determined as the median value of the three highest low concentration samples (total number of the replicates n = 60) yielding in more than 95% positive scores above LoB.

The calibration curve was determined with cTnI concentrations of 5–200,000 ng/L. Each concentration was run in three replicate strips. The calibration curve was run in each testing day in parallel with patient samples to assess daily variations. The curves were fitted by using linear regression for logarithmic values. The patient samples (n = 262) were assayed with the UCNP-LFIA procedure in triplicates and the sample concentrations were calculated against the calibration curve run at the same time with the evaluated set of samples.

Parallelism was assessed by serial dilution of three LiH plasma samples with endogenous cTnI concentrations of 9365, 7329 and 3060 ng/L (measured with the in-house well-based assay). Each sample was diluted up to 1/81 with blank LiH plasma pool and each dilution was measured in three replicates.

### Statistical analysis

Statistical analyses were performed with IBM SPSS Statistics 21 (SPSS Inc., Chicago, IL, USA). Noted p values were 2-tailed, and all p values < 0.05 were considered statistically significant. Normality of the distribution of continuous variables was determined by the Shapiro–Wilk test and visual inspection. For the clinical samples, assay descriptive and Spearman’s rank correlation coefficients were calculated. Passing and Bablok regression analysis (MedCalc Software, MedCalc Software Ltd., Ostend, Belgium) was used when studying linear regression between the UCNP-LFIA and the reference method. The linearity of sample dilutions was assessed with linear regression analysis.

## Supplementary Information

Supplementary Information.

## Data Availability

The datasets generated and analyzed during the current study can be made available by the corresponding author upon reasonable request.
