# Supplementary material for: Sensitive and quantitative detection of cardiac troponin I with upconverting nanoparticle lateral flow test with minimized interference
Source: Sci Rep. 2021 Sep 21;11:18698. doi: 10.1038/s41598-021-98199-y (PMC8455528; doi:10.1038/s41598-021-98199-y)
Supplement: Supplementary file 1 — Supplementary Information. [file 41598_2021_98199_MOESM1_ESM.docx]

*
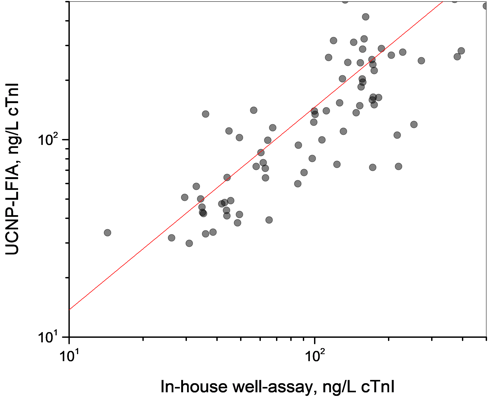
*

A

***Fig. S1.*** *Method comparison between the UCNP-LFIA and the in-house well-based reference assay (n=188). A) Correlation of the assays and B) Bland-Altman analysis of agreement. The relative difference is calculated as UCNP-LFIA concentration subtracted by in-house well-based assay concentration divided by mean concentration. The mean difference (39.4%) is presented with a solid line and the 95% limits of agreement (from -55.1% to 134%) are shown with dashed lines. A close-up showing the correlation between the developed LFA and the in-house well-based assay within the cTnI concentration range of less than 100 ng/L.*
